# Extraction socket preservation using a collagen plug combined with platelet-rich plasma (PRP): A comparative clinico-radiographic study

**DOI:** 10.34172/joddd.2020.028

**Published:** 2020-06-17

**Authors:** Numaan Nisar, Kumar Nilesh, Mushtaq Ishaq Parkar, Prashant Punde

**Affiliations:** ^1^Department of Oral and Maxillofacial Surgery, School of Dental Sciences, Krishna Institute of Medical Sciences, Karad, India

**Keywords:** Alveolar ridge, Collagen, Graft, Platelet-rich plasma

## Abstract

**Background.** Alveolar bone remodeling after tooth loss results in reduced ridge dimensions in horizontal and vertical planes. To prevent this, various authors have proposed different ridge preservation techniques. A collagen plug is a novel material that has shown promising results in preserving the alveolar bone. PRP has also yielded favorable outcomes in wound healing and promoted osteoinduction and osteoconduction

**Methods.** Thirty patients of both sexes with an age range of 30–18 years requiring bilateral extraction of teeth with similar tooth root anatomy in the maxilla or mandible were included in the study. The extraction of teeth was carried out atraumatically. The patients’ arches were randomly divided and labeled as the test or control sides. Bone width was measured on both sides. A collagen plug, with PRP, was placed, and the extraction socket was sutured on the test side. The control side was just sutured. A baseline RVG was taken to record the apico-coronal height. The patients were recalled after 10 days for suture removal and evaluation of wound healing. Parameters were re-evaluated at three and six months postoperatively. The data were subjected to t-test and one-way ANOVA.

**Results.** The height of the crestal bone on the grafted side was more when compared to the non-grafted side three and six months after tooth extractions, and the difference was statically significant (P<0.001). No statistically significant difference was seen in the width of the alveolar bone three and six months after tooth extraction (P>0.05).

**Conclusion.** Collagen and PRP provided reasonable socket preservation as simple and inexpensive options as compared to other materials.

## Introduction

Preservation of the alveolar ridge following tooth extractions represents a challenge in everyday clinical practice.^1^Tooth extraction, which might be carried out for several reasons, is a traumatic procedure resulting in the loss of alveolar bone. Alveolar bone remodeling that occurs after tooth loss leads to a decrease in bone volume (vertical and horizontal).^1,2^ Numerous studies have demonstrated that following tooth extraction, bone remodeling takes place during wound healing, which leads to bone loss (up to 40–60% of bone loss in height and width in three months).^3^The buccal bone is more prone to resorption when compared to the lingual. The reason behind this phenomenon is the presence of a higher amount of bundle bone in the buccal cortex.^1,3^ This excessive resorption of buccal cortical bone causes significant aesthetic problems.^1^ Resorption rates vary among individuals. It even fluctuates for the same person within the same periods. There is a significant difference in the resorption rates between the maxilla and the mandible, with sockets in the mandible being resorbed up to four times faster than the maxilla.

Without any intervention, 40–60% of the total alveolar bone volume is lost in the first two to three years after tooth extraction.^1,4^Maintenance of an adequate bone volume is a necessity for the success of dental implants, both functionally and esthetically. Many operators graft the extraction site after tooth extraction using various commercially available bone graft materials to achieve adequate bone volume at the time of implant placement.^5^ Grafted extraction sites have shown better bone volume (loss of width of <2 mm and a loss of height of <0.5 mm) when compared to non-grafted extraction sites (loss of width: 2–6 mm and loss height: 1 mm).^1-3^

Socket preservation is an excellent method to solve the problem of low bone volume and increased rate of bone loss after tooth extraction. “Socket preservation is a procedure in which bone graft materials are placed in the extraction site at the time of tooth extraction.”^6^ Socket preservation has unpredictable results as the procedure is technique sensitive.^1^ A wide variety of bone graft materials are available for grafting the extraction sockets, including autologous grafts, allogenic materials, and xenografts. These bone grafts have been useful in socket preservation. However, the choice of material depends on the technique employed for socket preservation.^1^

After placing the graft in the extraction socket, it is mandatory to cover it with a membrane. Various authors have proposed different methods of covering the graft material to prevent the ingress of soft tissue. The materials/techniques which have been used are collagen membranes, primary soft tissue coverage, a free gingival graft, or a connective tissue graft, and placement of a collagen plug.^1-3^ Barrier membranes have shown good results in ridge preservation;^1^ however, the drawback of this technique is that it requires primary soft tissue closure, which causes repositioning of the mucogingival junction, displacement of the keratinized mucosa towards the crestal region, and an increase in postoperative swelling and discomfort. There is also a risk of secondary exposure, which could jeopardize the outcome of the grafting procedure.

Various other techniques have evolved over the years, including the socket seal surgery technique and Bio-Col technique, to counter the drawbacks of flap advancement.

“Socket plug” technique^1,7,8^ is a term used by a few authors to include all the variations of methods of socket preservation. This technique is based on four steps; 1) atraumatic tooth extractions, 2) a conservative flap design, 3) placement of appropriate biomaterials, and 4) suturing.

An innovative addition in the field of dentistry is platelet-rich plasma (PRP), which was introduced approximately two decades ago. It is a concentrated source of platelet-derived growth factors. These growth factors help accelerate wound healing and are used for tissue engineering.^5^ PRP, along with a collagen plug, provides a scaffold for periosteal cells in vitro, which is suitable for applications in bone tissue engineering.^4,5^ Because of the regenerative potential of PRP, it has good efficacy in peri-implant bone regeneration. Thus, PRP delivers a high concentration of growth factors at the site of bone augmentation, and in combination with the collagen plug, it promotes osteoinduction, osteoconduction, and osteogenesis.^1^

Considering the importance of hard and soft tissue healing at extraction sites and exploration of the role of PRP and collagen plugs, the present study was designed to evaluate the efficacy of collagen plugs (KOLSPON PLUG) with PRP in post-extraction sockets and compare it with the non-grafted site using a split-mouth study design.

## Methods

This study was undertaken in the Department of Oral and Maxillofacial Surgery, School of Dental Sciences, KIMSDU, Karad, India, after approval of the Institutional Ethics Committee. Thirty patients of both genders, requiring extraction of teeth bilaterally with the same tooth root anatomy either in the maxilla or mandible, who were willing to participate and sign an informed consent form, were selected for the study. Patients having teeth associated with acute periapical pathology, medical conditions which compromised bone healing, medically compromised patients, and patients with a history of head and neck irradiation were not included in the study

Before starting the surgical procedure, PRP was prepared using a double centrifugation method;^9^ 2 mL of blood was drawn from the cubital vein of the patients using a 3-mL disposable syringe. The blood was then transferred to a Vacutainer bulb containing an anticoagulant (sodium citrate) and mixed thoroughly. The Vacutainer bulb was placed in the centrifuge machine and was centrifuged at 2500 rpm for 10 minutes (soft spin); the soft spin yielded about 1.3 mL of the middle layer (buffy coat), which was withdrawn from the centrifuged blood and transferred to a plain Vacutainer blub (without anticoagulant) which was again placed in a centrifuge machine and centrifuged for 10 minutes at 3400 rpm (hard spin). Approximately 1 mL of PRP was prepared and collected in a small steel bowl and activated with 0.1 mL of calcium gluconate just before placing in the extraction socket.^9^

**Figure 1:**
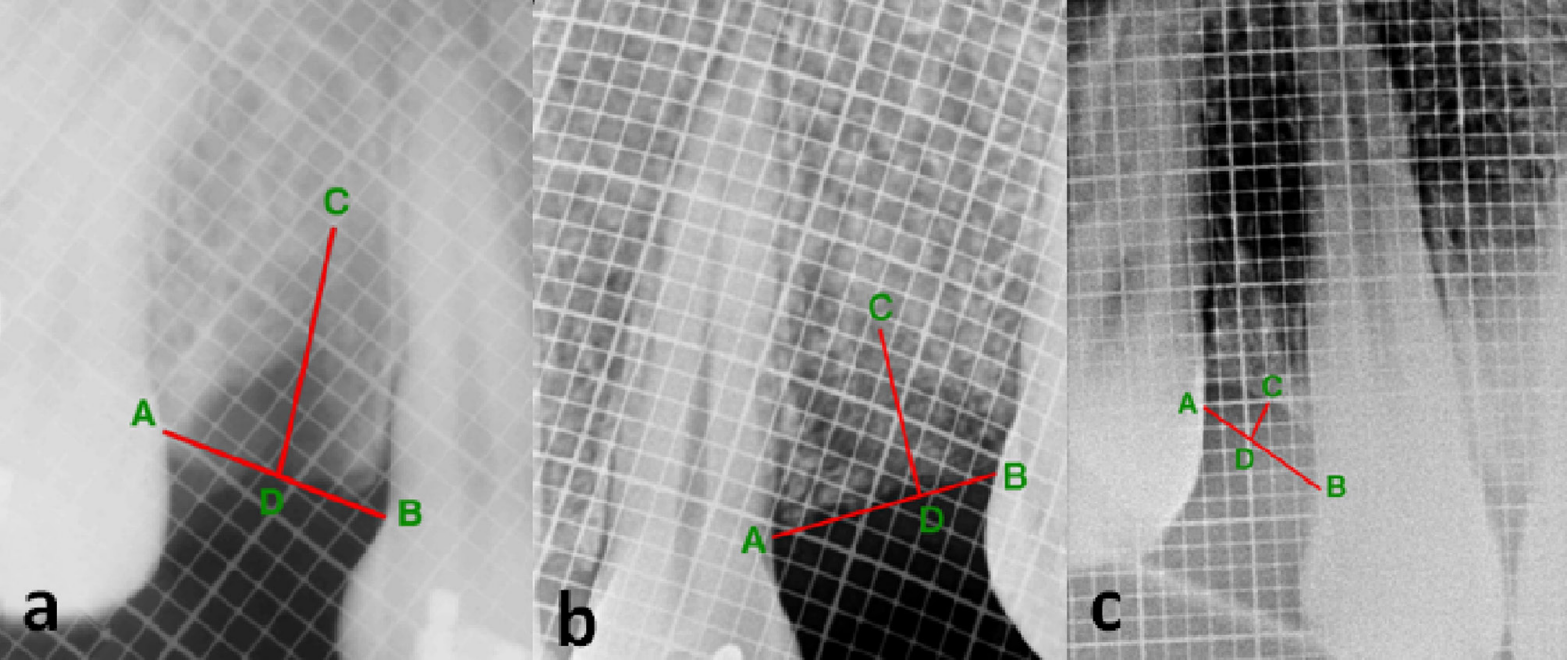
Postoperative radiograph of the grafted site at (a) baseline, (b) 3 months, and (c) 6 months, showing the measurement of alveolar bone height.

The height of the alveolar bone was assessed by the radiovisiography (RVG) (Carestream Health Inc., NY, USA) technique. A connecting line (AB) was drawn from the cementoenamel junctions (CEJ) of adjacent teeth on the mesial and distal aspects of the extraction socket. The lowest position on the alveolar ridge defect was marked as point C. A line was drawn from C, keeping it perpendicular to the line AB. Point D was marked on line AB, at the intersection of the line drawn from C. The distance of CD was measured at 3- and 6-month follow-up visits. A radiopaque millimeter-graduated grid (Bluedent India Private Ltd., Chennai) was used to standardize the assessment of the height of the alveolar bone radiographically across the extraction socket postoperatively. A decrease in the length of CD signified an increase in bone height and vice-versa (Figure 1).

**Figure 2:**
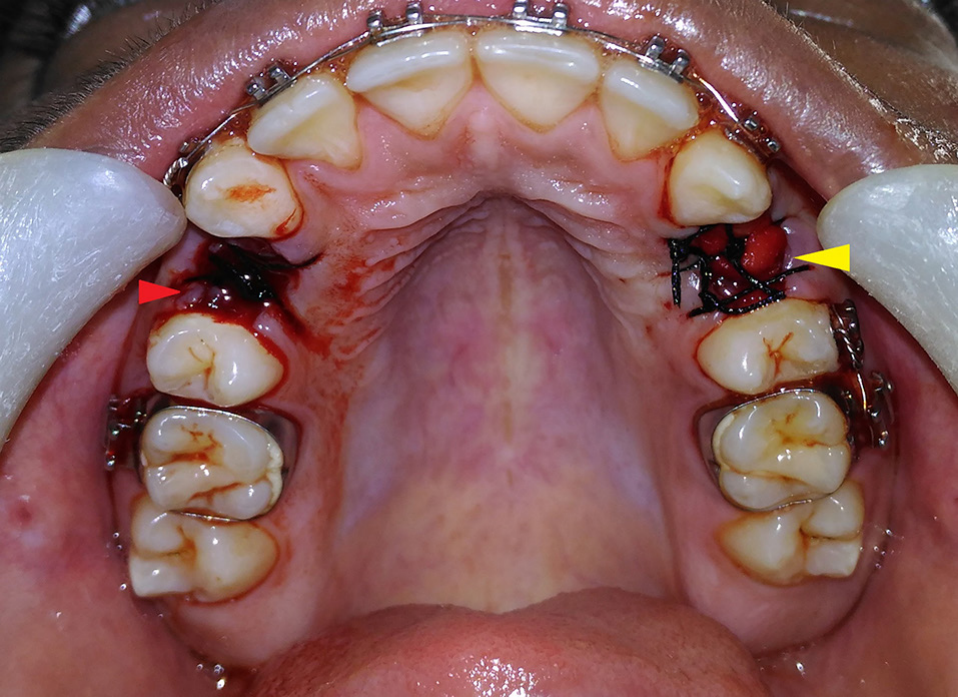
Postoperative photograph showing sutured grafted and non-grafted sites. [Patient’s right side, non-grafted site (red arrow), and left side, grafted site (yellow arrow).

All the procedures were carried out under strict aseptic conditions. Following the administration of local anesthesia (2% LOX containing 2% lignocaine with 1:200000 adrenaline by Neon Laboratories Ltd.), the free gingival margin was elevated as conservatively as possible. The teeth were luxated using elevators and extracted atraumatically with forceps. After extraction, the sockets were carefully curetted. The patients’ arches (either maxillary or mandibular) were divided into quadrants and randomly labeled as test side and control side (in patients having even serial numbers, the right quadrant was labeled as the test side, and in patients with an odd serial number, the left quadrant was labeled as the test side). A collagen plug (KOLSPON PLUG) was inserted up to the crestal level in the extraction socket of the test side after saturating it with activated PRP and sutured into position using 3-0 silk sutures in the simple interrupted pattern. On the control side, the extraction socket was just sutured using 3-0 silk sutures in a simple interrupted pattern without placing any graft material (Figure 2). The width of the alveolar socket at the extraction site was measured using bone calipers at three levels (crestal, mid-root, and apical), immediately after extraction and after 3 and 6 months. A digital radiograph (RVG) was taken in combination with a radiopaque millimeter-graduated grid to evaluate the baseline bone height immediately after extraction and after 3 and 6 months. The extraction of the tooth and radiographic assessment were carried out by two different operators. The patients were given routine antibiotics and analgesics (Amoxicillin tablets, 500 mg TID, and Diclofenac sodium tablets, 50 mg BD for three consecutive days) and recalled 10 days after the surgery for suture removal and evaluation of soft tissue healing according to Landry, Turnbull, and Howley index.^20^

### Statistical analysis

An unpaired t-testwas used to assess the mean length of line CD (to evaluate the height of crestal bone) and the width of the alveolar bone between grafted and non-grafted extraction sites at baseline, and at 3- and 6-month postoperative intervals. One-way ANOVAwas used to compare the mean length of line CD at three time intervals, i.e., at baseline and 3- and 6-month intervals.

## Results

### Soft tissue healing assessment (Table 1)

**Table 1:** Soft tissue healing assessment

	**1st day**		**10th day**	
	**Grafted sockets**	**Non-grafted sockets**	**Grafted sockets**	**Non-grafted sockets**
**Tissue color >50% red**	29	29	3	3
**Bleeding on palpation**	29	29	0	0
**Granulation tissue**	0	0	29	29

The extraction sockets were evaluated for soft tissue healing on the 10th postoperative day based on Landry, Turnbull, Howley healing index (tissue color, bleeding on palpation, granulation tissue); there was no statistically significant difference in soft tissue healing when grafted and non-grafted sites were compared (P>0.05).

### Assessment of bone height (Table 2)

**Table 2:** Assessment of the mean length of line CD (in mm) over a period of time

		**Bone height**	**t-statistic**	**P-value**
**Baseline**	**Non grafted Site**	4.000 ± 0.802	0.642	0.523
	**Grafted Site**	4.138 ± 0.833		
**Three months**	**Non grafted Site**	4.931 ± 0.842	5.56	<0.001*
	**Grafted Site**	3.741 ± 0.842		
**Six months**	**Non grafted Site**	5.310 ± 0.795	8.57	<0.001*
	**Grafted Site**	3.483 ± 0.829		

RVG was taken at baseline and 3- and 6-month postoperative intervals to assess the height of the crestal bone. The unpaired t-test was applied to compare the height of crestal bone in grafted and non-grafted sites. The difference was statistically significant at three months (the length of CD at grafted sites: 3.741±0.842 mm; the length of CD at non-grafted sites: 4.931±0.842 mm; P<0.001) and six months (the length of CD at grafted sites: 3.483±0.829 mm; the length of CD at non-grafted sites: 5.310±0.795 mm; P<0.001) after tooth extraction. The result was suggestive of greater height of the crestal bone at grafted sites as compared to non-grafted sites.

One-way ANOVA was used to compare the lengths of CD in grafted and non-grafted sites at baseline and 3- and 6-month postoperative intervals. There was a statistically significant difference in the height of the crestal bone between grafted (P=0.01) and non-grafted (P<0.001) extraction sites. At grafted sites, the mean length of the line CD at baseline was 4.138 mm, with 3.741 mm after three months and 3.483 mm after six months. At non-grafted sites, the mean length of CD was 4.000 mm at baseline, with 4.931 mm after three months and 5.310 mm after six months.

### Assessment of bone width (Table 3)

**Table 3:** Assessment of clinical bone width (in mm) over a period of time

		**Crestal**	**Mid-root**	**Apical**
**Baseline**	**Non-grafted site**	8.862 ± 1.060	9.034 ± 1.180	9.069 ± 1.132
	**Grafted site**	8.431 ± 1.100	8.586 ± 1.150	8.724 ± 1.192
	**t-statistic**	1.520	1.465	1.130
	**P-value**	0.134	0.148	0.263
**Three months**	**Non-grafted site**	7.672 ± 1.088	8.241 ± 1.154	8.621 ± 0.942
	**Grafted site**	7.655 ± 1.119	8.138 ± 1.093	8.655 ± 1.143
	**t-statistic**	0.059	0.35	0.125
	**P-value**	0.953	0.727	0.901
**Six months**	**Non-grafted site**	6.672 ± 1.020	7.828 ± 1.197	8.345 ± 0.814
	**Grafted site**	7.190 ± 1.072	7.914 ± 1.001	8.586 ± 1.086
	**t-statistic**	1.882	0.298	0.958
	**P-value**	0.065	0.767	0.342

The width of the alveolar bone was measured (in mm) using bone calipers at three levels, i.e., at crestal, mid-root, and apical regions. There was no statistically significant difference in the width of bone at any of the levels when compared at grafted and non-grafted sites (P>0.05).

The variables were subjected to one-way ANOVA. There was a statistically significant reduction in crestal bone width in grafted sites (P<0.001), whereas in the non-grafted sites, there was a statistically significant reduction in bone width in the crestal (P<0.001) and mid-root levels (P<0.001).

## Discussion

Teeth are extracted frequently in the oral surgery clinic. It is often necessary to extract teeth when they become non-restorable, which might be due to various disease processes such as chronic periodontitis, extensive carious lesions, periapical pathology, and root fractures due to trauma or any other cause.^10,11^ Tooth extraction, even when carried out with great caution and following an atraumatic procedure, leads to the loss of alveolar bone.^1^ This loss of bone occurs in height as well as in width, and the bone loss is very rapid in the first 6–12 months following tooth extraction.^12-14^ It has been demonstrated that up to 40% and 60% of bone height and width, respectively, might be lost in the first 6–12 months after extraction.^14^

Over the past decades, various techniques have been tried with variable success rates to preserve the alveolar bone. Different procedures and techniques of alveolar ridge preservation include regenerative techniques using autografts, allografts, and xenografts with or without a collagen plug, resorbable/non-resorbable membranes, immediate implants, and use of PRP.^1,4,6,8^

Regenerative technique makes use of different materials which are placed in the defect created in the alveolar bone after tooth extraction.^15^ These materials include autologous bone, allogenic bone material, and xenograft.^7,8,11,17^ These materials can then be covered with a membrane or autologous tissue to prevent their loss.^8,18^ These materials have shown promising results in preserving the alveolar bone.^18^ Bone loss in grafted sites has been reported to be <0.5 mm in height and <2 mm in width; however, non-grafted sites exhibit up to 1 mm of loss of height and 2–6 mm in width within one year after tooth extraction.^1,2,17^

A novel technique in socket preservation is to use a collagen plug, which is a cylindrical-shaped collagen sponge tailored to fit in the extraction socket.^1,3,5^ This material fits snugly in the extraction socket as a scaffold and serves as a chemotactic agent for fibroblasts.^1^ It additionally helps in hemostasis at the extraction site.^1^ Over the past decade, several variations of socket preservation techniques have been tried, where bone graft materials and collagen have been used in various combinations, known as “socket plug technique.”^1^ This technique has shown to be reliable, with predictable outcomes in terms of alveolar bone preservation.

Various clinicians have extensively used immediate implant placement as a technique for alveolar ridge preservation with variable results.^16,20^ The success of this modality depends on the type of implant used, quality of bone, patient systemic factors, presence of any deleterious habits like smoking, and the area of alveolar bone where the implant is placed.^16^ Some authors have reported that the peri-implant defects heal by the formation of connective tissue instead of bone-to-implant contact.^17^ However, recent studies have shown comparable bone levels around implants placed immediately after extraction and implants placed in healed extraction sockets.^20^ However, immediate implant placement after extraction in anterior maxilla should be considered cautiously as this region has a higher rate of implant failure.^12^ Another disadvantage of alveolar bone preservation with implants is that they are costly and might not be a feasible option for many people who cannot afford them.

Platelet-rich plasma is a source of growth factors (platelet-derived and transforming growth factor-beta) that is obtained by centrifuging and concentrating platelets by gradient density centrifugation.^21^The use of PRP in dentistry was introduced about two decades ago. The reason for including this novel material in dentistry was the concentration of various growth factors, such as platelet-derived growth factors (PDGF), epithelial growth factors (EGF), vascular endothelial growth factors (VEGF), and transforming growth factor-beta (TGF-β) in PRP, which are more than four times those in whole blood.^9^ PDGF has been postulated to promote soft and hard tissue regeneration. Growth factors like EGF and VEGF promote soft tissue healing by inducing epithelial proliferation, and neo-vascularization.^9,22^ Growth factors like PDGF promote the proliferation of bone marrow and osteoblasts and help in osteoid formation,^20^ thus promoting bone healing. TGF-β is another essential growth factor present in PRP, which belongs to a superfamily of growth factors of which BMPs are also a part. These growth factors, similar to PDGF, promote cellular proliferation, stimulate matrix production, and guide differentiation towards cartilage or bone.^9,21,22^

The present study used a collagen plug combined with PRP as a graft material for socket preservation. No previous study has utilized this combination for socket preservation. The rationale behind using this combination was that it combines the soft and hard tissue healing capabilities of PRP with collagen and makes it available in the extraction socket, which is not possible when PRP is used alone (as PRP after activation forms a gel-like mass which is difficult to contain in alveolar sockets). Collagen, on the other hand, provides a scaffold in the extraction socket and helps in osteoconduction, and when combined with PRP, it helps hold it within the alveolar socket.^1,4^ Combining both these materials seemed logical to test the properties of both materials in socket preservation. This combination was safe as PRP was obtained by centrifuging patients autologous blood^9^, and the collagen plug used in this study was derived from highly purified type 1 collagen of fish origin, which is antigenically inert and does not induce hypersensitivity reactions.^19^ It is relatively cheaper in comparison to other socket preservation techniques.

Various authors have proposed different protocols for the preparation of PRP. Some authors suggest a single spin method, while others suggest two spin methods with different rpms.^9,21,22^ However, the number of spins depends on the type of centrifuge used.^22^ Some centrifuges which are specially designed for the preparation of PRP complete the procedure in a single cycle.^21,22^ The protocol which was followed in the present study included two cycles. The first spin, i.e., soft spin, was carried out at 2500 rpm for 10 minutes, followed by the second spin, i.e., hard spin, at 3400 rpm for 10 minutes, which is consistent with other studies.^9,21,22^

The parameter which was assessed in the soft tissue profile was gingival healing. It exhibited no statistically significant difference when a comparison between the test and control sites was made (P>0.05). This again could be attributed to the placement of sutures in the test and control sites, in addition to the selection of patients who were free from periodontal or periapical pathology in the test and control sites.^1^

For the assessment of hard tissue profile in grafted and non-grafted sites, the height of the crestal bone and the width of the alveolar bone were evaluated. Various authors have also evaluated the same parameters to measure the amount of available bone for implant placement.^1,3,7^ In the present study, the height of the crestal bone was measured radiographically (using RVG), using the long cone technique, consistent with previous studies.^2^ A radiopaque millimeter-graduated grid was used along with RVG to standardize the radiograph, to account for any distortion, and to help measure the height of the crestal bone. The distance between the two radiopaque squares in the grid was 1 mm.

In the present study, there was a statistically significant difference in bone height between the grafted and non-grafted sites at baseline and 3- and 6-month postoperative intervals (P<0.001), with more bone loss in non-grafted sites compared to grafted sites. The width of the alveolar bone was measured clinically with the help of bone calipers at three different levels (crestal, mid-root, and apical levels). There was a reduction in bone width after three and six months in both grafted and non-grafted extraction sites. The reduction in width in grafted sites was about 1 mm less than that in non-grafted sites. However, the difference was not statistically significant (P>0.05). Another difference that was observed when comparing grafted and non-grafted extraction sites was greater bone resorption in the mid-root region in non-grafted sites after six months. This resorption in non-grafted extraction sites was statistically significant compared to grafted sites. These findings are not consistent with previous studies as the literature suggests that after socket grafting, there is a significant difference in width between grafted and non-grafted sites, with grafted sites exhibiting significantly less bone resorption than non-grafted sites.^1,6,7^ In the present study, no significant difference was observed in bone width between grafted and non-grafted extraction sites. The reason behind this could be the use of interrupted sutures over the extraction socket, placed to prevent the graft from dislodging. This could have exerted some pressure over the crestal bone, causing resorption, which can be avoided by placing horizontal mattress or figure-of-eight sutures.

The socket plug technique, like every procedure, has some limitations as well. This technique cannot be applied in areas where the buccal plate of bone has fractured.^1,17^ In such areas, the bone graft material has to be supported by a barrier membrane, and the results are not predictable. Another disadvantage is that this technique is contraindicated in areas of acute infection because, in such cases, there can be a rapid dissolution of collagen sponge and failure of the graft material.^1,17,18^ Another factor that should be considered is to avoid or limit flap elevation^1,5^ at the extraction site as this limits bone resorption.^8,19^

The limitations of the study are its small sample size and the lack of the use of more precise methods of assessing bone volume, like CBCT.

## Conclusion

Socket grafting is an easy and predictable way of preserving the alveolar bone, which later leads to better prosthetic and aesthetic outcomes for the patients. The materials (collagen saturated with PRP) used in this study resulted in favorable outcomes in terms of preservation of bone height but did not show any significant results in terms of preservation of bone width. To conclude, a collagen plug combined with PRP should be considered as an economical and predictable option for socket grafting. Further in vivo studies must be conducted using collagen plugs and PRP, with a larger sample size to make sure of the ridge preservation potential of these materials.

## Acknowledgment

None.

## Authors’ Contributions

NN was theprincipal investigator. KN drafted and finally approved the manuscript. MP carried out proofreading and revised the manuscript. PP carried out the initial corrections of the manuscript.

## Funding

Self-funded project.

## Competing Interests

The authors declare no competing interests with regards to authorship and/or publication of this article.

## Ethical approval

The institutional ethics committee of Krishna Institute of Medical Science approved this study (Ref. No KIMSDU/IEC/03/2016, Protocal No. 2016-2017/100).
